# NOCTIS: open-source toolkit that turns reaction data into actionable graph networks

**DOI:** 10.1186/s13321-025-01118-w

**Published:** 2025-12-04

**Authors:** Nataliya Lopanitsyna, Marta Pasquini, Marco Stenta

**Affiliations:** Syngenta Crop Protection AG, Schaffhauserstrasse, 4332 Stein, AG Switzerland

**Keywords:** Reaction data mining, Chemical reaction networks, Open-source software, Synthetic route, Synthesis planning, Computer-aided synthesis design

## Abstract

**Background:**

Chemical reactions form densely connected networks, and exploring these networks is essential for designing efficient and sustainable synthetic routes. As reaction data from literature, patents, and high-throughput experimentation continue to grow, so does the need for tools that can navigate and mine these large-scale datasets. Graph-based representations capture the topology of reaction space, yet few open-source tools exist for building and querying such networks. To address this, we developed NOCTIS, an open-source toolkit for constructing and analyzing reaction data as graphs.

**Results:**

NOCTIS is an open-source Python package for building Networks of Organic Chemistry (NOCs) from reaction strings. It supports graph-based analysis, parallel processing of large datasets, and export to common Python formats (e.g., NetworkX, pandas). Built on Neo4j technology, it features a modular, extensible architecture with open-source dependencies. We also provide a companion plugin for exhaustive route enumeration. It traverses graph-encoded reactions to assemble all valid synthetic routes, helping prevent redundant exploration and supporting knowledge reuse in synthesis planning. The underlying algorithm is documented in detail along with its current limitations. Using the MIT USPTO-480k dataset (Adv Neural Inf Process Syst 30, 2017), we demonstrate the plugin’s route mining capabilities, analyze network connectivity, and assess synthetic trees.

**Conclusion:**

Built on LinChemIn (J Chem Inf Model 64(6):1765–1771, 2024), NOCTIS serves as an open and extensible toolkit for network-based reaction analysis and route mining, laying the groundwork for data-driven route design at scale. Future work will extend query capabilities and improve the efficiency of route extraction.

## Scientific contribution

NOCTIS provides a programmatic, query-driven framework for exploring large reaction networks beyond the limitations of traditional tabular data analysis. Synthetic routes are extracted via a built-in query invoking the plugin and can be used in downstream workflows. Its flexible architecture allows users to customize or extend workflows without modifying the underlying code, making it suitable for large-scale, automated analysis.

## Introduction

Advances in high-throughput experimentation [1–4], automated synthesis [5–8], increasingly supported by computational methods [9, 10] have impacted the generation of chemical reactivity data [11–17]. Chemical databases have consequently expanded both in size and in the level of detail they contain. At the time of writing, the Pistachio (2025Q2) dataset includes about 22 million reactions [18], Reaxys offers about 71 million reactions [19] and CAS Reactions exceeds 150 million reactions [20]. These numbers highlight the necessity of effective data management aligned with FAIR principles (Findable, Accessible, Interoperable, and Reusable) [21–24].

Traditionally, chemical reaction data have been stored [25–30], searched [31, 32], and analyzed [33–35] using tabular formats. However, these traditional approaches have limited capacity to represent the inherently complex and interconnected nature of chemical transformations [36]. To overcome this limitation, researchers have increasingly adopted more sophisticated data representations [37], shifting toward network-based structures, common in biological and biochemical sciences for metabolic pathways [38–40].

Chemical Reaction Networks (CRNs) [41, 42] and chemical knowledge graphs [43, 44] defined using rigorous ontologies [11, 45–47] have demonstrated their value by providing deep mechanistic and kinetic insights [48–50]. Network-based representations facilitate diverse applications, including synthesis planning [51, 52], identification of strategic chemicals [53, 54], route optimization focusing on cost, efficiency, and intellectual property considerations [55–58] and route mining [59]. Moreover, CRNs enable sustainable synthetic strategies through biomass valorization and waste repurposing [60–62].

At million scale, simple graph solutions built with standard language primitives become memory-bound and lack declarative, multi-user querying. In contrast, graph databases provide index-backed pattern matching, ACID-compliant persistence, and disk-based storage suitable for reproducible team workflows.

We therefore adopt a graph-database backbone and export selected subgraphs to Python for downstream analysis. Configuring and tuning such workflows are non-trivial, which contributes to the current adoption gap.

To address these needs, we present NOCTIS (Network Of Organic Chemistry: Transforming Information into Synthesis), an open-source Python package that converts reaction strings into a bipartite reaction–molecule graph persisted in a graph database. The package supports domain-specific and user-defined queries with export to common Python formats (e.g., pandas, NetworkX). In addition, it provides a practical route mining workflow with configurable bounds ( max_number_reactions, stop-node properties) implemented as a Java plugin. We demonstrate NOCTIS on the MIT USPTO-480k dataset [63]. Build and runtime figures and graph statistics are reported in the Results section. The additional materials in the supplementary information (SI) and the project repository enable reproducing the results discussed in the next section.

We present the data model and ontology in Sect. "Ontology and data model", followed by a brief implementation overview (Sect. "Implementation"). In Sect. "Route extraction" we introduce the Java route mining plugin (*Route Miner*). We then demonstrate large-scale graph construction and route mining capabilities on the MIT USPTO-480k dataset [63]. Finally, in Sect. "User workflows on the example of MIT USPTO-480k dataset" we include a comparison anaysis with to the PaRoutes dataset [64].

## Ontology and data model

**Fig. 1:**
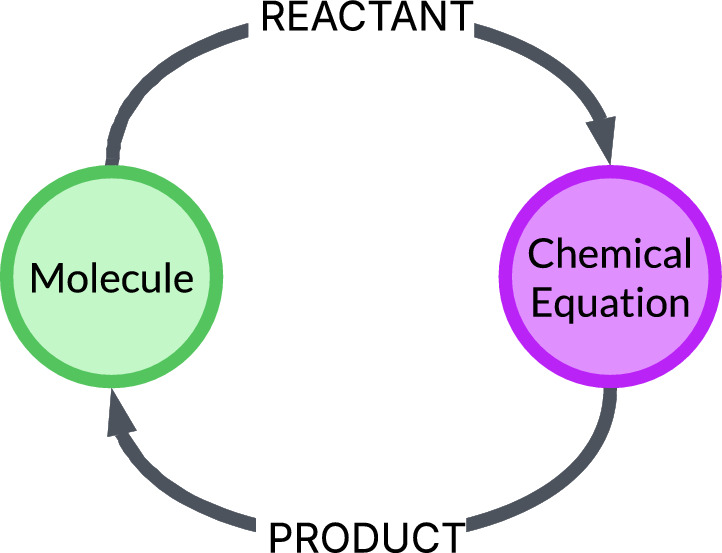
Depiction of a bipartite core graph schema. Molecule nodes (green) and reaction nodes (purple) are interconnected through PRODUCT-REACTANT relationships, illustrating how molecules serve as both products and reactants within various reactions

NOCTIS is built on the premise that reaction data can be represented as a network of molecule and reaction nodes connected by PRODUCT-REACTANT relationships as shown in Fig. 1. The LinChemIn [65] paper describes the bipartite schema adopted here. In this framework, all nodes and relationships are implemented as Python classes, with unique identifiers computed from their corresponding reaction string representations. These classes can host, besides the attributes necessary to the NOC, any arbitrary user-defined properties, thus allowing rich metadata annotation.

The bipartite model is the foundational schema in NOCTIS, and all analyses in this work, including route mining, operate on this core PRODUCT-REACTANT layer. The schema can be extended with user-defined node and relationship types. The minimal data model including reactants and products can be extended to include reagents and their specific role in the reaction, whenever available (see the SI 7.3). Future releases of the package will extend and simplify reagent inclusion in the NOC.

At its core, NOCTIS employs the DataContainer, a versatile class that encapsulates the results of graph queries. This container is capable of storing multiple records–each record comprising a collection of node and relationship objects–without imposing strict structural validation. This open-ended design enables users to extract and manipulate diverse graph structures, whether they be disconnected nodes, linear pathways, synthetic trees, or synthetic routes.

A synthetic route is defined as a directed graph that embodies a sequence of necessary and sufficient chemical transformations that are required to synthesize a target molecule from a number of starting material. The graph representing the route has one root (the target molecule) and all relationships are oriented toward it. Since each molecule in a route is the product of only one reaction, each Molecule node within a graph can have only one PRODUCT relationship with a ChemicalEquation node. A synthetic tree aggregates multiple synthetic routes converging on the same target, offering a comprehensive view of alternative synthesis strategies. It is important to highlight that a synthetic tree does not conform to the strict definition of a tree in graph theory; it is a directed graph with a root that may contain cycles.

The extraction of these graph patterns from an NOC leverages a combination of predefined custom user-defined queries. while the former are part of the code base, the latter can be implemented either in code or via YAML configuration files. In particular, a dedicated Java plugin for Neo4j supports the extraction of synthetic routes for a specified target, an invaluable capability for retro-synthetic analysis and automated synthesis planning.

Detailed information about the Python classes and graph schema is provided in Sect. "Implementation", while the custom query mechanisms are described in Sect. "Implementing user-defined queries with YAML". The route mining algorithm is presented in Sect. "Route extraction".

## Implementation

NOCTIS loads reaction data to graph databases, ensuring rigorous input validation while constructing a reaction networks from individual reaction data points. Its functionality is built around three core modules–*Data Architecture*, *Data Transformation*, and *Repository*–each addressing a specific aspect of data modeling, processing, and querying.

Leveraging Object-Oriented Design (OOD) and Domain-Driven Design (DDD) principles, NOCTIS accurately represents complex cheminformatics data while promoting modularity, maintainability, and scalability. This design simplifies the management of intricate chemical datasets and ensures each component remains reusable and extensible.

At the core is the *Data Architecture* module, which implements a typed data model representing chemical entities and their interrelationships as a graph. It provides strict type validation and immutability, enforces the schema, and packages query results for downstream use. This backbone ensures that ingestion, graph construction, custom schema extensions, and built-in queries all operate consistently on a single, well-specified representation.

The *Data Transformation* module covers preprocessing and export functionalities. In preprocessing, it ingests a list of reaction strings, CSVs, or pandas DataFrames and expands them into nodes and relationships ready for graph-database loading. This stage is parallelizable and may apply user-configurable validation (standardization, canonicalization of reaction strings, role assignment). On export, it transforms query results as Python objects (pandas DataFrames, NetworkX graphs, LinChemIn objects).

The *Repository* module implements the query layer, providing built-in queries for common tasks and support for user-defined queries via YAML templates. Built-ins include creating the graph database from scratch, transactional insertion of new graph objects, and route mining. Route mining is executed by a Java plugin, invoked through the built-in interface, and operates on the core PRODUCT-REACTANT layer. Query results are then exported via *Data Transformation* module.

**Fig. 2:**
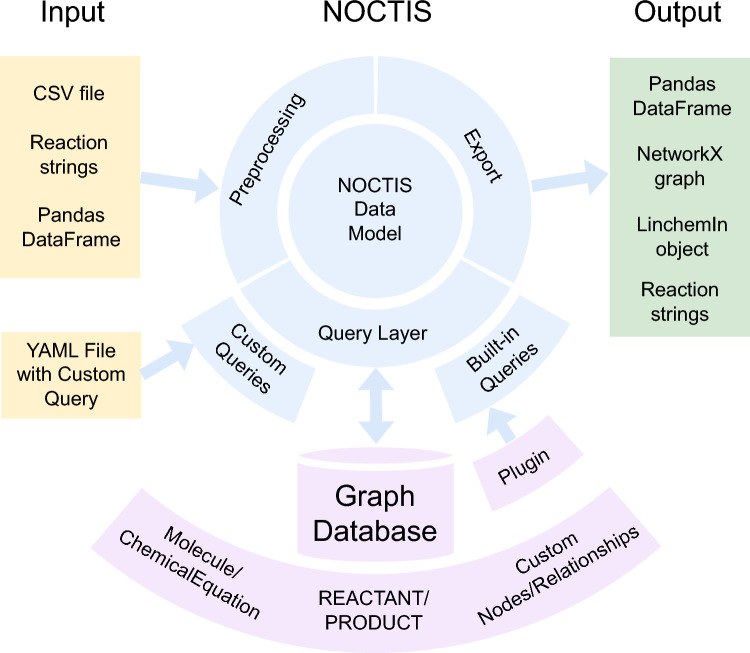
System overview of NOCTIS. Inputs (CSV, pandas DataFrames, reaction strings) pass through a parallelizable preprocessing stage *(Data Transformation module)* where an optional, user-configurable validation step (standardization, canonicalization, role assignment) may be applied. The NOCTIS data model *(Data Architecture module)* provides the typed backbone by defining nodes and relationships, enforcing the schema, and packaging query results. The query layer *(Repository module)* then builds and queries a persisted reaction–molecule graph in a graph database; within the database, duplicates merge by unique identifiers and custom schema elements (additional node/relationship types) can be added. The route mining plugin is invoked via built-in queries. Results are exported via the *Data Transformation module* to common Python formats (pandas tables, NetworkX graphs, LinChemIn objects) for downstream analysis

In Fig. 2, we provide an overview of the NOCTIS workflow and how the code components participate in it. Further implementation details about module internals 7.5, class diagrams 7.11, configuration options 7.4, YAML query templates 7.8, and other information 7.6, 7.7 are provided in the SI.

## Route extraction

In this section, we introduce the synthetic route extraction functionality implemented in NOCTIS. Its purpose is to identify all possible synthetic routes—from starting materials to the target—within a reaction network using only the core schema (Molecule and Chemical Equation nodes, along with PRODUCT-REACTANT relationships). The route mining algorithm–implemented as a Java plugin, referred to below as *Route Miner*, is accessible through a dedicated query that triggers the exhaustive enumeration of all viable synthetic routes.

This section begins with a detailed description of the route extraction algorithm implemented in *Route Miner*, highlighting how the plugin traverses reaction networks to construct complete synthetic routes. We then introduce a comprehensive test graph to evaluate the algorithm’s robustness against challenges typical of real-world reaction data. Finally, we discuss the exponential growth of route possibilities driven by branching at OR nodes and outline both current and future strategies for mitigating this combinatorial explosion, emphasizing the role of intelligent mining techniques in avoiding full enumeration.

Before introducing the algorithm, we first define the concept of an OR node, which is central to understanding its structure and logic.

**Fig. 3:**
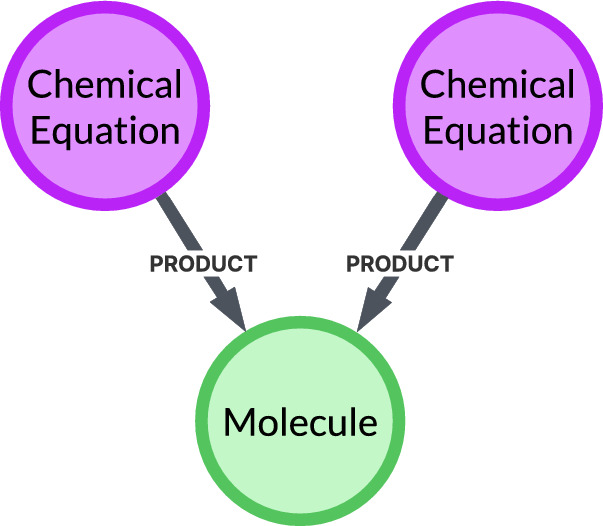
OR Node illustration. This diagram highlights a Molecule node receiving multiple incoming PRODUCT relationships from distinct reaction nodes, indicating that the molecule can be produced via multiple alternative reactions

***The Concept of*** **OR** ***Nodes*** A core concept in the route mining algorithm is the AND/OR semantics of nodes. In our bipartite graph, ChemicalEquation nodes have AND semantics: if a route contains a reaction, then all of its REACTANT neighbors must appear in that same route. Molecule nodes can exhibit either semantics. With a single incoming PRODUCT relationship they effectively have AND semantics—the producing reaction (and its required reactants) co-occur in the same route. With multiple incoming PRODUCT relationships they have OR semantics, because each incoming relationship represents an alternative provenance, and any given route may include at most one such PRODUCT relationship for that Molecule. Thus, an OR Molecule can be produced by more than one reaction, yielding distinct alternative synthetic routes to the same target (see Fig. 3 for an illustration of an OR node).

### Algorithm implementation

NOCTIS performs route extraction by invoking a Neo4j-backed Java plugin through the query layer to enumerate all synthetic routes from a specified Molecule target, under optional user-defined bounds (maximum number of reaction steps and stop constraints). The plugin traverses the core PRODUCT-REACTANT bipartite graph and returns the results in the NOCTIS data model (as DataContainer objects). The plugin is packaged as a JAR for Neo4j and can be called from Neo4j Desktop or programmatically via NOCTIS; installation and build instructions are provided in the SI 7.1 and the project repository. ***Design and Workflow***

The algorithm is designed to mine routes through reaction networks by decomposing the problem into two primary phases: subroute extraction and route assembly. In the first phase, the algorithm traverses the reaction network using a breadth-first search (BFS) starting from a unique root node. As it moves through the network, contiguous segments where only one reaction is possible for each molecule are collapsed into single entities which we call subroutes. Each subroute is defined by a root (where it starts) and a set of leaves (where the subroute terminates, typically at an OR node or a dead end). This phase produces a mapping from nodes to subroutes, isolating the decision points where multiple synthetic routes emerge.

The second phase, route assembly, uses the precomputed subroutes to build complete routes. Starting from one or more initial subroutes mapped to the target molecule, the algorithm iteratively extends partial routes by “stitching” together subroutes from the mapping. At each step, it examines the current pending leaves and uses an iterative expansion to explore all possible extensions. We also provide a pseudocode of the algorithm in the SI 7.9.

***Handling Cycles***

In the context of reaction networks, a cycle is a closed sequence of reactions that ultimately leads back to the original materials, allowing the process to repeat infinitely. This concept is central to catalytic processes and metabolic pathways, and could be something desirable to have and explore. However, cycles can create potential problems for a traversal algorithm, making it stuck in a loop. To avoid that, our algorithm addresses the cycles in the reaction network in two ways. During the subroute extraction phase, cycles are managed using a custom uniqueness filter. The filter ensures that within each path explored during BFS the relationships are unique by checking that no relationship appears more than once. This prevents the traversal from following cyclic paths repeatedly.

In the route assembly phase, the algorithm maintains a visited set during the traversal. This set keeps track of the nodes that have already been used as subroute roots in the current route. By doing so, the algorithm avoids reintroducing a node that has already been included, ensuring that each route is built by selecting subroutes that progress through the network without

### Test graph construction

To rigorously validate the scientific output of our route extraction algorithm, we constructed a comprehensive test graph consisting of 27 nodes and their associated relationships. It abstracts away all chemistry (no real reactions) and uses simple numeric indices for nodes. This test tree was deliberately designed to incorporate typical challenging cases that may occur in a real reaction network and is intended to serve as a standard benchmark for validating future route mining algorithms.

The test graph covers a wide range of challenging scenarios, including:

- **Cycles:** which can potentially cause infinite recursions if not handled correctly.
- **Double additions and multiple products:** representing cases where the same reaction step contributes to different synthetic pathways.
- **Common intermediates:** where a single molecule participates in multiple reactions.
- **Branching within a cycle:** which tests the algorithm’s ability to navigate complex network topologies.
- **Concurrent branching from a single node:** simulating scenarios where several synthetic routes from a single root can be obtained due to simultaneous branching along multiple paths.

Additionally, tests were conducted by initiating route mining from both the true root of the tree and from other nodes, as mining solely from the root may not capture cases where the root molecule lacks branching.

The Cypher code used to construct this test graph, along with the correct answers for the tests, is provided in supplementary materials and the visualization of the test tree can be found in the SI 7.10.

### Exponential growth of synthetic routes and mitigation strategies

When two routes are merged into a single synthetic tree and share intermediates, those shared molecules become OR nodes if generated by distinct reactions, creating new ways to combine existing reactions into alternative routes that differ from the originals. Consequently, even small synthetic trees can explode into an enormous number of possible routes, sometimes numbering in the millions. In such cases, because the route mining plugin enumerates all routes reachable in the tree, full enumeration becomes infeasible, and the user may receive no results within practical limits.

To address this, NOCTIS provides user-defined bounds that still return useful results under combinatorial growth. One option is to mark molecules with a stop property (e.g., startingMaterial) to halt expansion at designated Molecule nodes. Another is to impose a maximum traversal depth, capping the length of any linear sequence of reactions in a route. For example, projecting startingMaterial property onto specific molecules prevents further expansion beyond them. Alternatively, a limit on the longest linear sequence yields routes of manageable length and supports incremental exploration. If no bounds are specified, the algorithm expands until it reaches true leaves (molecules with no incoming PRODUCT relationships).

Looking ahead, we anticipate a shift toward smart route mining–methods that avoid exhaustive enumeration. By leveraging intrinsic network properties and integrating tools like language models or graph-based learning, it becomes possible to score and prioritize routes without generating all possibilities. As more curated records of executed synthesis pathways become available, hybrid approaches combining historical data with real-time graph exploration will play a central role in guiding efficient and meaningful synthesis planning.

## User workflows on the example of MIT USPTO-480k dataset

This section serves as a tutorial, exemplifying some of the most common workflows supported by NOCTIS. NOCTIS supports bulk ingestion from structured CSV files, incremental updates to an existing graph from Python objects, built-in queries for graph manipulation and analysis including route mining, and user-defined queries. Here we focus on built-in queries and YAML-driven custom queries, demonstrated on the MIT USPTO-480k dataset [63]. The remaining workflows, including schema definition and configuration  7.2,  7.3, bulk ingestion details 7.6, CSV structure and requirements 7.6, preprocessing options 7.4, performance and scaling 7.6, and incremental updates 7.7, are documented in the SI. We assume the graph database is already instantiated and accessible. We refer the reader to the official Neo4j documentation. We provide a concise setup guide in the SI Section 7.1. All examples shown here are available as a Jupyter notebook in the project repository, and supporting diagrams illustrating class usage and interactions can be found in the SI Section 7.11.

***Graph database creation*** NOCTIS can construct a graph database from scratch by processing CSV files in parallel. During loading, the graph database merges duplicates by unique identifiers, so deduplication happens at import time after preprocessing rather than as a separate step inside NOCTIS. Optional validation (standardization, canonicalization, and role reassignment) with LinChemIn [65] helps ensure a chemically consistent network. By default, two reactions are considered duplicates if their standardized and canonical reaction SMILES produced by the configured preprocessing are identical. If validation and preprocessing are disabled, duplicates are detected by exact string equality of the input reaction fields. On the MIT USPTO-480k dataset, preprocessing with validation enabled completes in approximately 30 min when executed in parallel using 5 workers (20 threads total) on a machine equipped with a 12th Gen Intel Core i9-12900 H CPU, 64 GB DDR5-4800 RAM, and a 512 GB NVMe SSD. Disabling validation reduces processing time to about 6 min. A scaling plot and additional configuration details are provided in the SI 7.6.

For the analyses that follow, we created a graph from 479,035 reaction strings of USPTO-480k, which includes the original train, test, and validation splits. The resulting database contains 627,508 Molecule nodes and 453,339 ChemicalEquation nodes, with 468,714 PRODUCT and 797,716 REACTANT relationships. No reaction strings failed validation. The reduction in distinct reaction nodes relative to the input reflects cases that, after validation and role reassignment, were mapped to the same unique ChemicalEquation identifier.

### Built-in queries for graph data manipulation and analysis

NOCTIS provides built-in queries that enable straightforward data manipulation and analysis. Users can quickly access all available queries via Neo4jRepository.info(). Among these queries are standard Cypher queries, Python-based queries that dynamically generate Cypher strings, and a query for mining synthetic routes powered by *Route Miner*. Please ensure that the plugin is activated beforehand, as instructed in the SI 7.1. Below, we show how to call any built-in query with NOCTIS on the example of the get_routes query:
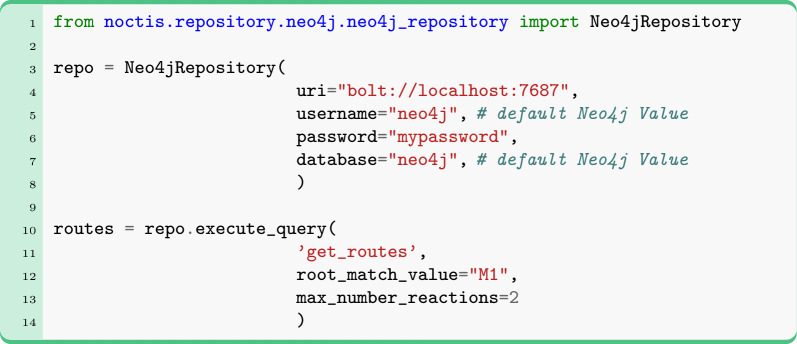


Queries that retrieve graph objects from the database return a DataContainer object. This DataContainer holds a list of GraphRecord objects, with each record corresponding to a single match from the executed query. Using its instance methods, the DataContainer can be transformed into various formats: a list of reaction strings, two pandas DataFrames (one for nodes and one for relationships), a NetworkX object (combining all data), or a Syngraph (LinChemIn object).
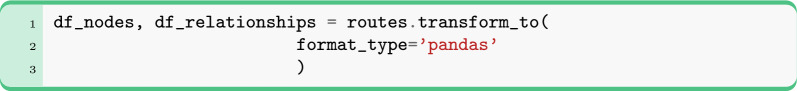


During transformation, users can choose whether to keep the separation by record IDs by setting the with_records_id parameter to true or false. If true (the default behavior), each node and relationship in the pandas DataFrames will include a record_id column; for other formats, nodes and relationships will be separated into distinct objects per record (e.g., a list of lists for reaction strings, a list of Syngraphs, or a list of NetworkX objects) rather than being merged into a single object.

***Mining Synthetic Routes for Paracetamol from MIT USPTO-480k Data***

We chose paracetamol as an example of a well-studied target to demonstrate NOCTIS’s route mining capabilities on the MIT USPTO-480k dataset. By limiting the search to sequences of up to four reactions, we uncovered 99 distinct routes (Table 1). Notably, paracetamol’s synthetic tree includes 9 OR nodes (intermediates with multiple synthesis alternatives), each with 2–10 alternatives–enough to yield nearly 100 routes even at this modest depth. Manually identifying so many routes would be cumbersome without specialized tools like NOCTIS.

**Table 1:** Synthetic route metrics for paracetamol mined from MIT USPTO-480k using NOCTIS

Metric	Value	Avg. Value
Target Compound	Paracetamol	
SMILES	CC(=O)Nc1ccc(O)cc1	
Number of Routes	99	
Longest Sequence	1–4	3.6
Number of Steps	3–7	5.8
Number of Branches	2–6	4.8
Number of OR nodes*	9	
N Products per OR node*	2–10	4.2

With the asterisk, we marked the properties which attribute to the synthetic tree of paracetamol

In this illustrative use case, we focused on extracting all valid route objects, rather than analysing further their similarity, highlighting the tool’s ability to systematically map every match in the reaction graph. While NOCTIS efficiently traverses these possibilities, the number of potential routes can soar beyond $\sim 10{,}000$ for more complex targets, pushing the limits of practical computation.

***Comparison of NOCTIS Routes to PaRoutes Dataset***

To evaluate the performance of the route extraction algorithm implemented in NOCTIS, we compared its output against the PaRoutes dataset, published by AstraZeneca and made available by Genheden et al. [64]. We treat this comparison as a sanity check rather than a head-to-head benchmark: PaRoutes assembles per-patent, local route trees [64, 66], whereas NOCTIS mines routes from a global, multi-source reaction graph. Accordingly, our questions are whether NOCTIS can recover or supersede PaRoutes routes (by topological overlap/containment) and where differences arise from construction assumptions rather than algorithmic failure. While PaRoutes is based on a more recent version of the MIT USPTO-480k dataset, our work uses the earlier version curated by MIT [63], which introduces challenges for direct comparison due to differences in reaction coverage. To address this, we enriched our MIT-derived reaction network by incorporating reactions corresponding to the PaRoutes routes, creating a hybrid graph that enables meaningful cross-dataset evaluation.

Our evaluation had two primary goals: (1) to demonstrate that multiple alternative routes can be mined from a reaction network–something not feasible without *Route Miner*; and (2) to demonstrate how NOCTIS operates on graphs that merge multiple datasets. We initially selected 11 target molecules that appear in both the n1 and n5 benchmark sets of PaRoutes. Each of these targets is associated with a single synthetic route in PaRoutes–mostly linear, with one exception containing two branches.

However, due to the high complexity of some targets, route mining for five of them exceeded practical computational limits, and we excluded those cases from further analysis. We continued with the remaining six targets.

To ensure a fair comparison, we aligned NOCTIS’s stopping criterion with the maximum linear sequence length of the corresponding PaRoutes routes by setting the max_number_reactions parameter accordingly. It is important to note that since the starting materials used in PaRoutes do not always appear as such in our dataset, NOCTIS sometimes expanded routes further than the PaRoutes ones–continuing to explore possible precursors of those “starting materials.”

We assessed the results using several metrics, including the number of alternative routes identified, the number of reactions and branches per route, and whether the mined routes were strict supersets of the corresponding PaRoutes route.

**Target1:** The PaRoutes route is fully recovered, and no alternative routes are discovered. None of the reactions in the PaRoutes route are connected to other reactions in the MIT USPTO-480k, so NOCTIS retrieves exactly one route, which matches the PaRoutes route.

**Target2:** One of the three routes found by NOCTIS does not contain any of the reactions from the PaRoutes route. This indicates that NOCTIS discovered an alternative synthesis route based solely on reactions from the MIT USPTO-480k subset.

**Target3 and Target5:** All NOCTIS routes are supersets of the PaRoutes route, meaning that all mined routes include all reactions from the PaRoutes route and expand beyond them.

**Target4:** One reaction in the PaRoutes route has an alternative transformation in the MIT USPTO-480k set. As a result, only three out of six routes mined by NOCTIS share five out of six reactions with the PaRoutes route.

**Target6:** This is the most complex case. NOCTIS discovers 270 distinct routes, all variations of the PaRoutes route with extended branches beyond the original starting materials. The branching diversity comes exclusively from exploration upstream of the PaRoutes-defined starting materials, illustrating how NOCTIS explores the full precursor space.

Table 2 summarizes the results for six selected targets defined in Table 3. “Shared Reactions” refers to the number of reactions common between the PaRoutes route and mined routes. “Not PR Superset” indicates how many mined routes are strict supersets of the corresponding PaRoutes route.

**Table 2:** Comparison of NOCTIS-mined synthetic routes with routes from PaRoutes dataset for selected targets

Target	Number of Routes	Shared Reactions	Steps PR/NOCTIS	Longest Sequence PR/NOCTIS	Number of Branches PR/NOCTIS	Not PR Superset
Target1	1	7	7/7	7/7	0/0	0/1
Target2	3	0–7	7/1–11	7/7	0/6	1/3
Target3	3	6	6/8–9	6/6	0/5	0/3
Target4	6	5–6	6/7–8	6/6	0/3	3/6
Target5	11	7	7/9–10	7/7	0/5	0/11
Target6	270	7	7/10–13	6/6	2/4	0/270

**Table 3:** Mapping of target identifiers to SMILES strings

Target	SMILES
Target1	COc1ccc(CN2CC(C(C)(C)S(=O)(=O)c3cccc(C(F)(F)F)c3)CC2=O)cc1
Target2	COc1ccc(-c2ncc(F)c(N(C)CCCOc3ccc4c(ccn4CC(=O)O)c3)n2)cc1
Target3	N#Cc1cc(Cl)c(C(=O)c2c[nH]c3cnccc23)c(Cl)c1
Target4	Cc1ccc(CC(=O)O)c(O)c1
Target5	CN(C)C(=O)c1cc(F)cc(C(C2CN(C(c3ccc(Cl)cc3)c3cccc(C#N)c3)C2)C(C)(C)F)c1
Target6	CCC(O)(/C=C/c1ccc(C(CC)(CC)c2cc(C)c(-c3ccc(CC(=O)O)c(F)c3)c(C)c2)cc1C)CC

### Implementing user-defined queries with YAML

Although NOCTIS has a collection of built-in queries, it also allows users to create their own queries through YAML files. All queries in NOCTIS can be of three types: retrieve_graph (read-only, returning data as DataContainer objects), modify_graph (write-only, modifying the database contents), and retrieve_stats (read-only, returning query statistics as pandas DataFrames). When a custom query is defined, the query type has to be provided explicitly. Below is an example of a YAML file for a retrieve_graph query (for other examples see SI 7.8):
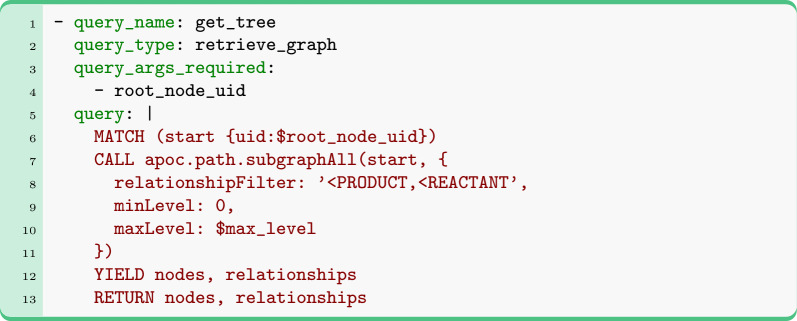


Executing this custom query:
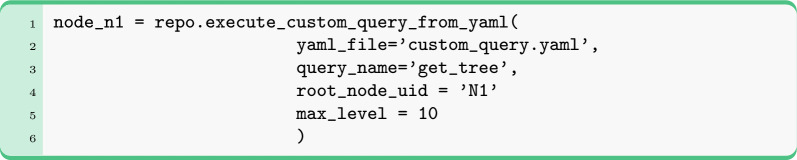


***Custom Query Statistics and Route Estimation*** We ran custom queries on the MIT USPTO-480k dataset to extract key statistics, which are summarized in Table 4.

**Table 4:** Summary of custom query statistics on the MIT USPTO-480k dataset

Metric	Value
Root molecules	308,010
Leaf molecules	188,614
Avg. reactants per reaction	1.76
Avg. products per reaction	1.04
Product-to-molecule ratio	2.53
OR nodes	12,347

Total nodes: 627,508 Molecule nodes and 453,339 ChemicalEquation nodes. Total relationships: 468,714 PRODUCT and 797,716 REACTANT relationships

The result provides a high-level view of the connectivity and complexity within the reaction network built on the MIT USPTO-480k. The high number of root molecules suggests a broad base of target products, while the number of leaf molecules indicates many starting materials. The average number of reactants per equation (1.76) reflects the prevalence of bimolecular and unimolecular reactions. The products-to-molecules ratio hints at moderate reuse of molecular nodes, while the number of OR nodes (12,347) highlights substantial potential for route branching. These statistics support the observed difficulty of route estimation and reinforce the need for structure-aware methods like NOCTIS to manage the underlying combinatorial complexity.

The queries were implemented using YAML configuration files and executed via a provided Jupyter notebook. Both the YAML files and the notebook are included with this work, serving as an example of how users can define and run their own queries to extract statistics or perform custom analyses over reaction graphs.

## Conclusions

We introduced NOCTIS, an open-source Python package for constructing and analyzing chemical reaction networks from reaction strings. It supports customizable data models, Cypher-based queries, and seamless integration with Python tools such as pandas and NetworkX, all backed by a Neo4j graph database. Its Object-Oriented and Domain-Driven Design principles promote modularity and make community-driven extension straightforward.

The implementation is organized across three modules–Data Architecture, Data Transformation, and Repository–whose roles are illustrated through workflows on the MIT USPTO-480k dataset. *Route Miner* enables the extraction of all possible synthetic routes, addressing the challenge of route recovery in complex networks. Comparison with the PaRoutes dataset highlighted the algorithm’s completeness and scalability while exposing the difficulties of interpreting route multiplicity without guidance.

Future developments will focus on expanding query capabilities, introducing route scoring strategies, and improving performance. NOCTIS sets the foundation for data-driven route design and supports the cheminformatics community with an open, extensible platform for navigating complex synthetic spaces.

## Supplementary Information

Additional file 1.

## Data Availability

All source code, documentation, and example workflows that support the findings of this study are openly available under the MIT License at https://github.com/syngenta/noctis (core framework) and https://github.com/syngenta/noctis-route-miner (route extraction plugin). Reproducibility scripts and step-by-step instructions to recreate all figures and tables are available in the jupyters directory of the main repository. NOCTIS (Core Framework) Programming language: Python ≥3.9 Other requirements: dynaconf pyyaml neo4j ≥5.0.0 networkx pandas ≥2.0.0 pydantic ≥2.0.0 tqdm dask dask[dataframe] dask[distributed] rdkit linchemin ≥3.0.0 NOCTIS Route Miner Plugin Programming language: Java Other requirements: Java Development Kit (JDK) 17 Gradle (or use the included Gradle wrapper) Neo4j Kernel 5.14.0 JUnit Jupiter 5.8.2 (testing) Neo4j Harness 5.14.0 (testing) Mockito Core 4.3.0 (testing) Database Requirements To run a Neo4j database locally, Neo4j Desktop is required.
