# Supplementary material for: NOCTIS: open-source toolkit that turns reaction data into actionable graph networks
Source: J Cheminform. 2025 Dec 4;18:6. doi: 10.1186/s13321-025-01118-w (PMC12798089; doi:10.1186/s13321-025-01118-w)
Supplement: Supplementary file 1 — Additional file 1. [file 13321_2025_1118_MOESM1_ESM.pdf]

7 Supplementary Information

7.1 Neo4j Desktop Setup Guide

This guide describes how to set up Neo4j Desktop from scratch for chemical data processing, including installing the APOC plugin, adding a custom plugin, and configuring the database for handling large datasets. This guide was written in 2025Q2, so please check the official guide for latest details:
https://neo4j.com/docs/desktop-manual/current/

1. Install Neo4j Desktop

Download and install the latest version of Neo4j Desktop from the official website.

2. Create a New Project and Database

- Open Neo4j Desktop.
- Click `+ New` →`Create Project`
- Inside your project, click `Add Graph` →`Local DBMS`.
- Name your local database management system (DBMS), set a password, and click `Create`.
- A single DBMS can host multiple databases. These databases will share the same plugins and configuration settings, but each one contains its own isolated graph. By default, the primary database is named `neo4j`.

3. Configure the Database for Large Datasets

If planning to load more than ~1 million nodes or relationships, adjust memory settings as follows:

1. Right-click your DB →`Open Folder` →`DBMS`.
2. Open the file `conf/neo4j.conf`
3. Alternatively, through Neo4j Desktop: right-click your DB →`...`→`Settings`
4. Modify the settings:

```
dbms.memory.heap.initial_size=2G
dbms.memory.heap.max_size=2G
dbms.memory.pagecache.size=2G
```

4. Install and Configure APOC Plugin

- In the `Plugins` tab of your database, click `Install` next to `APOC`.
- To enable file import via APOC, create a file named `apoc.conf` in the `conf` directory and add:

```
apoc.import.file.enabled=true
apoc.import.file.use_neo4j_config=true
```

- For newer versions of Neo4j Desktop, also set in `neo4j.conf`

```
dbms.security.procedures.unrestricted=apoc.*
dbms.security.procedures.allowlist=apoc.*
```

- Ensure your import files are located in: `<DB-FOLDER>/import/`
- Optionally, if you want to be able to load files from anywhere in your file system, make sure to comment in `Settings` (or `neo4j.conf`)

  ```
  # server.directories.import=import
  ```

  and set in `apoc.conf`

  ```
  apoc.import.file.enabled=true
  apoc.import.file.use_neo4j_config=false
  ```

5. Add Custom Plugin

- Place the plugin `.jar` file in: `<DB-FOLDER>/plugins/`
- Check compatibility with your Neo4j version.
- For newer versions of Neo4j Desktop, also set in `neo4j.conf`

  ```
  dbms.security.procedures.unrestricted=apoc.*,noctis.*
  dbms.security.procedures.allowlist=apoc.*,noctis.*
  ```

- To set up the java plugin for route mining – `noctis.route.miner` – follow the instructions on https://github.com/syngenta/noctis-route-miner.

6. Restart the Database

Restart your database in Neo4j Desktop to apply the configuration changes.

Checklist

- Neo4j Desktop installed
- Database created
- APOC installed and configured
- Custom plugin added
- Memory and import settings updated (if needed)
- `apoc.conf` created
- Database restarted

7.2 Defining the Graph Schema

In NOCTIS, nodes are merged by unique identifiers while a declarative graph schema controls how relationships are created and expanded. A default minimal schema is provided with two node labels—`ChemicalEquation` and `Molecule`—and two relationship types—`PRODUCT` and `REACTANT`—derived from the reaction strings. If this default meets your needs, you do not need to supply an explicit schema.

Naming conventions (canonical)

We adopt Neo4j's recommended style [67]: *CamelCase* for node labels (e.g., `ChemicalEquation`, `Molecule`) and *SCREAMING_SNAKE_CASE* for relationship types (e.g., `PRODUCT`, `REACTANT`, `FROM_SOURCE`). Digits and underscores are allowed.

Schema structure and mutability

The schema is defined with four top-level maps: `base_nodes`, `base_relationships`, `extra_nodes`, and `extra_relationships`. Keys inside `base_nodes` and `base_relationships` are fixed contract identifiers used by NOCTIS (`chemical_equation`, `molecule`, `product`, `reactant`) and must not be renamed. Values are the actual Neo4j labels and relationship-type strings and may be customized (e.g., you can change `"Molecule"` to `"Compound"` or `"PRODUCT"` to `"MY_PRODUCT"`).

All relationships are directional. Within each relationship entry, `type`, `start_node`, and `end_node` are required; the latter two must reference keys defined in `base_nodes` or `extra_nodes`.

Programmatic construction

You may build a schema from a Python dict or from a YAML file:

```python
from noctis.data_architecture.graph_schema import GraphSchema

gs = GraphSchema.build_from_dict(
                            {'extra_nodes':
                                {'molecule':'ExtraMolecule',
                                'chemical_equation':'ExtraChemicalEquation'
                                }
                            }
                            )

# gs = GraphSchema.build_from_file(
#                           file_path = 'schema.yaml',
#                           file_format = 'yaml'
#                           )
```

Examples of default and customized schemas are given in Section 7.3.

7.3 Graph Schema example

This section shows snippets of default and custom graph schema that conform to the rules in Section 7.2. Remember: keys inside `base_nodes` and `base_relationships` are fixed (`chemical_equation`, `molecule`, `product`, `reactant`); their values (labels and relationship types) are user-configurable. By default, these values are populated from the NOCTIS settings file at `~/noctis/settings.yaml`.

```python
# Default graph schema

{
            "chemical_equation": settings.nodes.node_chemequation,
            "molecule": settings.nodes.node_molecule,
        }
{
            "product": {
                "type": settings.relationships.relationship_product,
```

```
                "start_node": "chemical_equation",
                "end_node": "molecule",
            },
            "reactant": {
                "type": settings.relationships.relationship_reactant,
                "start_node": "molecule",
                "end_node": "chemical_equation",
            }
```

```
# Custom graph schema

{
    "base_nodes": {
        "chemical_equation": "ChemicalEquation",
        "molecule": "Molecule"
    },
    "base_relationships": {
        "product": {
            "type": "PRODUCT",
            "start_node": "chemical_equation",
            "end_node": "molecule"
        },
        "reactant": {
            "type": "REACTANT",
            "start_node": "molecule",
            "end_node": "chemical_equation"
        }
    },
    "extra_nodes": {
        "source": "SourceNode"
    },
    "extra_relationships": {
        "from_source": {
            "type": "FROM_SOURCE",
            "start_node": "source",
            "end_node": "chemical_equation"
        }
    }
}
```

Notes. (i) Relationship entries must include `type`, `start_node`, and `end_node`. (ii) `start_node` and `end_node` reference the keys (e.g., `molecule`, `chemical_equation`, `source`), not the label strings. (iii) All relationships are directional; edge direction determines traversal semantics used by NOCTIS.

7.4 Preprocessing parameters: defaults and options

The table 5 below describes the parameters used during the CSV preprocessing step. The parameter names mirror those used by the `Dask` library for parallel processing

and pandas for serial processing, where applicable. The fields inp_chem_format and out_chem_format define the chemical format of the input and output and support the following values: "smiles", "smarts", "rxn_blockV2K", and "rxn_blockV3K". Validation (validation=True) includes canonicalisation and standardisation of the chemical structures. The output_folder defines where the output files are stored, while tmp_folder is used to save intermediate temporary files during parallel processing. The parameter delete_tmp controls whether these temporary files are deleted after processing is completed. The prefix can be set to prepend a custom string to all output filenames prepared for import. Parameters such as delimiter, lineterminator, and quotechar allow customization of CSV formatting. The parallel flag enables or disables parallel processing. The parameter blocksize determines the size (in kilobytes) of each chunk when reading the file in parallel using Dask, while chunksize and nrows are used during serial processing to define the number of lines to read per chunk and the total number of rows to read, respectively.

**Parameter**	**CSV File**		**Python Object**	**Default Value**
	**Parallel**	**Serial**		
inp_chem_format	✓	✓	✓	"smiles"
out_chem_format	✓	✓	✓	inp_chem_format
validation	✓	✓	✓	True
output_folder	✓	✓	-	"./output"
tmp_folder	✓	✓	-	output_folder+"/tmp"
delete_tmp	✓	✓	-	True
prefix	✓	✓	-	None
delimiter	✓	✓	-	","
lineterminator	✓	✓	-	None
quotechar	✓	✓	-	"" "
parallel	✓	✓	-	False
blocksize	✓	-	-	600000 Kb
chunksize	-	✓	-	10000 lines
nrows	-	✓	-	None

Table 5 PreprocessorConfig Parameters and Their Applicability

7.5 Implementation Details

Data Architecture Module

The Data Architecture Module establishes the foundational structures for representing chemical entities and their interrelationships as a graph. At its core are Python classes—Node, Relationship, GraphRecord, DataContainer, and GraphSchema—all built on Pydantic's BaseModel [68] for robust type validation, immutability, and explicit schema definitions.

The Node and Relationship classes encapsulate chemical data and directional interactions, with nodes identified by unique identifiers and relationships defined by

their start and end node uids. The `DataContainer` aggregates query outputs as collections of `GraphRecord` objects, where each `GraphRecord` corresponds to a single query match.

Central to maintaining consistency is the `GraphSchema` class, which defines the graph's structural blueprint and validates custom schema elements. It ensures that all custom relationships correspond to defined node labels and supports the Data Transformation Module by guiding the expansion of input data into nodes and relationships. Additionally, `GraphSchema` verifies that the graph database schema remains unchanged during custom query execution and automatically uses correct node labels and relationship types in built-in queries.

Data Transformation Module

The Data Transformation Module streamlines the conversion of diverse chemical data inputs—ranging from CSV files and Pandas DataFrames to lists of reaction strings—into standardized, graph-compatible formats, either as CSV files for database upload or as `DataContainer` objects. Central to this module is the `Preprocessor` class, which leverages Factory and Template Method design patterns to harmonize data transformation across various input types. Users can fine-tune preprocessing through configurable parameters such as input/output chemical formats, parallelization settings, batch sizes, and validation preferences (see more in the SI 7.4). With support for parallel execution via Dask or serial processing to handle files in segments, the module efficiently manages large datasets without overwhelming system memory. Performance results are presented in Figure 4. Robust CSV header validation ensures compliance with expected formats, and any erroneous reaction strings are isolated and logged, enabling users to review anomalies post-processing. Additionally, the module facilitates reverse transformations by exporting internal `DataContainer` objects into popular Python formats—including Pandas DataFrames, NetworkX graphs, and LinChemIn-compatible objects—via the `ChemDataGeneratorFactory`.

Repository Module

The Repository Module acts as an abstraction layer between NOCTIS and the underlying Neo4j graph database, handling database-specific operations transparently and efficiently. It simplifies database interactions by managing sessions, transaction scopes, and query execution. Queries are categorized clearly into three types: `retrieve_graph`, `modify_graph`, and `retrieve_stats`, each with explicit operational modes and output formats. Built-in queries provide common functionalities, while custom queries defined through YAML files enable users to extend capabilities tailored to their specific research needs. Furthermore, the module's modular design permits contributors to easily integrate additional database systems or implement custom query functionalities without disrupting existing features, supported by well-defined interfaces and error-handling strategies. In the SI section 7.8, we describe in more details how to add custom queries to NOCTIS.

7.6 Initial Bulk Upload

NOCTIS enables users to construct a Graph Database from scratch by processing structured CSV files. One key advantage of using NOCTIS for this task is its ability to efficiently graphify large volumes of reaction data in parallel. During this process, NOCTIS automatically removes duplicates and optionally performs validation and sanitisation using the LinChemIn [2], ensuring the resulting graph is both consistent and chemically meaningful. By default, two reactions are duplicates if their standardized, canonical reaction SMILES (after the configured preprocessing) are identical; if validation/preprocessing is disabled, duplicates are detected by exact match of the input reaction strings.

CSV File Structure and Requirements

Bulk data uploads to the graph database require a CSV file structured with each row representing entities related to a single chemical reaction. The relationships are not provided explicitly but generated automatically based on the schema. Each row must include a reaction string within a mandatory column labeled `ChemicalEquation.smiles` or `ChemicalEquation.smarts`, supporting SMILES- and SMARTS-based reaction notations. Molecule nodes are generated automatically from these reaction strings and, therefore do not support additional properties defined in the CSV. The CSV header must adhere strictly to the format `Label.property_name`, and all nodes except molecule nodes may be assigned arbitrary properties. If there is a column named not following the pattern, it will be ignored, and a warning will be raised. Unique identifiers (uids) are essential for nodes—automatically computed from reaction strings for chemical equations but must be explicitly provided for user-defined nodes. The uid should start with one to three letters and followed by a sequence of numbers. If users provide their own uids for chemical equation nodes, they are going to be stored as a property with a name `user_uid`. An example of a correctly formatted CSV is provided below:

```
ChemicalEquation.smiles, ChemicalEquation.property, Src.uid, Src.name
A.B>>C,  value1, SID1, 'Internal'
E.D>>B,  value2, SID2, 'Patent'
G.F>>C,  value3, SID2, 'Patent'
```

Preprocessing and Data Ingestion

Preprocessing settings can be set from a YAML file or alternatively can passed directly to the method at run time, allowing users to specify parameters such as input/output chemical formats, parallelization options, batch sizes, and validation preferences (see more in the SI 7.4). Validation of reaction strings is optional; if a reaction string cannot be processed, the overall workflow continues, and the failed strings can later be found in a CSV file `{prefix}_failed_strings.csv`; if some rows contain empty reaction string, the absolute index of the row can be found in `{prefix}_empty_strings.csv`. All the indices start from 1, where 1 is the row of the header. Preprocessing can run in parallel or serially (in batches or fully processing the file), generating intermediate CSV files, which format adheres to required by APOC procedure format, in `tmp_folder` for

each serial or parallel partition. All the final files are saved in the `output_folder` – organised by node and relationship type – ready for database import:

```python
from noctis.data_transformation.preprocessing.data_preprocessing import
    Preprocessor

preprocessor = Preprocessor(schema = gs)

preprocessor.set_config_from_yaml(
                            file_path = 'preprocessor_config.yaml'
                            )

### Preprocessing of a csv file
preprocessor.preprocess_csv_for_neo4j_parallel(
                            input_file=INPUT_FILE,
                            prefix='USPTO',
                            )
```

After preprocessing, users instantiate the repository, ensuring that the APOC plugin is installed and file loading enabled. Database uniqueness constraints must be created before executing the bulk upload. Behind the `'import_db_from_csv'` is used `apoc.import.csv` procedure. If it meets the same reaction defined more than once within initial CSV input file, it will create nodes properties based on the first appearance and will ignore all the following, such as defined by `ignoreDuplicate=true` flag in Neo4j.

```python
from noctis.repository.neo4j.neo4j_repository import Neo4jRepository
# Default credentials are defined during NOCTIS configuration step
# in settings and secrets YAML files.

repo = Neo4jRepository(
                            uri="bolt://localhost:7687",
                            username="neo4j", # default Neo4j Value
                            password="mypassword",
                            database="neo4j", # default Neo4j Value
                            schema = gs
                            )

repo.create_constraints()
### Can be dropped with repo.drop_constraints()
### Or shown with repo.show_constraints()

### Bulk upload from csv
repo.execute_query(
                            query_name = 'import_db_from_csv',
                            prefix = 'USPTO'
                            )
```

Preprocessing Performance Evaluation

Large volumes of reaction data are recommended to import to a Graph DB from a CSV file. For the MIT USPTO-480k dataset containing about 480,000 reactions, parallel preprocessing with validation enabled took approximately 30 minutes on a machine with the following hardware specifications: a 12th Gen Intel Core i9-12900H processor, 64GB DDR5-4800 RAM, and a 512GB NVMe SSD, while disabling validation reduced processing time to about 6 minutes. These benchmarks were obtained using 5 workers and total 20 threads. Figure 4 illustrates how preprocessing time scales with different dataset sizes.

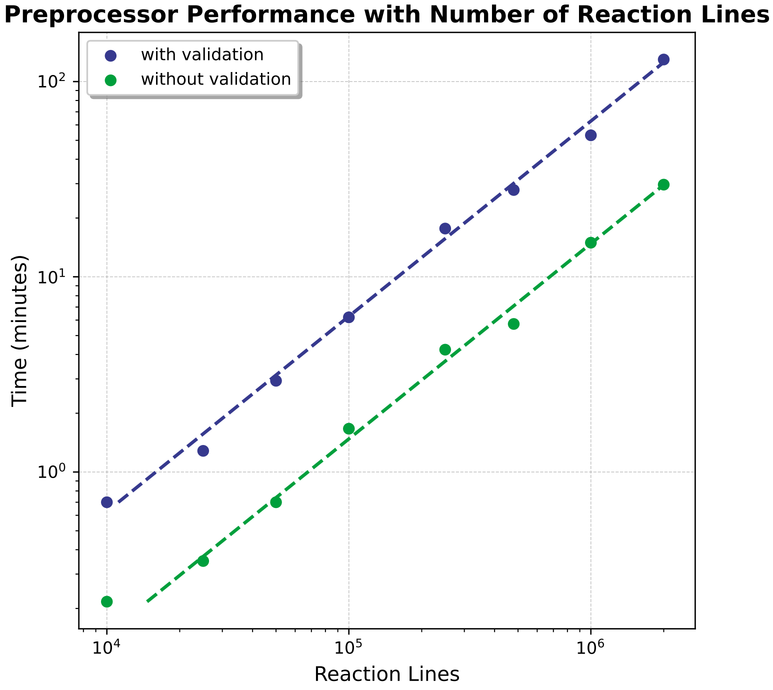

Fig. 4 Scaling performance of preprocessing times for reaction datasets of different size. The figure compares parallel preprocessing times with validation.

7.7 Incremental Updates

For smaller, incremental updates, Python objects can be used, including lists of reaction strings and Pandas DataFrames, provided they adhere to the same CSV format rules. Like CSV preprocessing, the workflow doesn't fail when encountering unparsable reaction strings; instead, these can be retrieved using a preprocessor method `get_failed_strings`. When processing a list of reaction strings, NOCTIS automatically generates only base schema nodes and relationships, without additional properties except for reaction strings. The preprocessor generates a `DataContainer` object, which can be directly uploaded:

```
### Preprocessing of a list of reaction strings
list_of_smiles = ['N.O>>C','T.I>>S']
data_container = preprocessor.preprocess_object_for_neo4j(
                        data=list_of_smiles,
                        data_type = 'reaction_string'
                        )

failed_strings = preprocessor.get_failed_strings()

### We assume that repository is already instantiated
### repo = Neo4jRepository( ... )

### Transactional upload from python object
repo.execute_query(
                        'load_nodes_and_relationships',
                        data = data_container
                        )
```

7.8 Queries: defining custom built-in query and YAML query

Query types

NOCTIS supports three query types that must be declared for both built-in and YAML-defined queries: `retrieve_graph` is *read-only* and returns a `DataContainer` of `GraphRecords` representing the retrieved subgraph/routes; `modify_graph` is *write-only* (executed in a write transaction) and returns a Python list of plain records describing the changes applied (not `GraphRecords`); `retrieve_stats` is *read-only* and returns a pandas `DataFrame` with aggregated metrics. The repository enforces the transaction mode (read vs. write) and post-processing based on `query_type`.

Custom built-in query

To add a new built-in query, users must define a subclass of the `AbstractQuery` base class and register it using the `@Neo4jQueryRegistry.register_query()` decorator. Each query must include a unique `query_name` and a `query_type` (`retrieve_graph`, `modify_graph`, or `retrieve_stats`). Lists of required and optional arguments can be declared via `query_args_required` and `query_args_optional`. If `parameters_embedded` is set to `False`, the query should include Cypher placeholders

(e.g., $node_uid), and arguments are injected at runtime. The query string can either be assigned directly to the query class attribute, or constructed dynamically within a _build_query() helper method that sets the query instance attribute to a single string. When parameters_embedded is set to True, parameters are meant to embedded directly into the query string, which should then be constructed as a list of strings inside _build_query(). For this type of query, each parameter case should be defined as an instance variable of the class. In both cases, if _build_query() is used, the get_query() method must be overridden to trigger _build_query(). The query_type determines how the result is post-processed and which data object is returned.

Custom query from YAML

Custom queries are defined using YAML and must explicitly declare all input arguments under query_args_required. All arguments referenced within the Cypher query must be labeled using the $ symbol (e.g., $smiles).

It is not necessary to reload or re-instantiate the code when modifying the YAML query file. The system reads and executes the query definitions dynamically at runtime.

```yaml
- version: 1.0
- date: 2025-05-04

- query_name: count_nodes
  query_type: retrieve_stats
  query: |
    MATCH (n)
    RETURN count(n)

- query_name: get_node_by_smiles
  query_type: retrieve_graph
  query_args_required:
    - smiles
  query: |
    MATCH (n {smiles:$smiles})
    RETURN n

- query_name: delete_all_nodes
  query_type: modify_graph
  query: |
    MATCH (n)
    DETACH DELETE n

```

7.9 Route Mining Algorithm Pseudocode

The route mining engine is implemented as a Neo4j plugin (JAR) and exposed to NOCTIS via a registered query. The NOCTIS query class GetRoutes is registered in the internal query registry Neo4jQueryRegistry and dispatches to the plugin's stored procedure noctis.route.miner. Conceptually, the procedure accepts: (i) a

root Molecule identifier; (ii) optional bounds (maximum number of reaction steps in any linear segment); and (iii) optional stop constraints (a node-property name that terminates traversal).

Traversal operates only on the core bipartite layer using the relationship types PRODUCT and REACTANT between node labels Molecule and ChemicalEquation. In NOCTIS, relationship types and node labels of the core schema are fetched from GraphSchema and passed to the plugin. So, if the user modifies the names of the core schema objects, this will be propagated to the plugin automatically.

The procedure returns routes as a stream of records; each route is converted by NOCTIS into a GraphRecord (nodes + relationships) and aggregated in a DataContainer (list of records).

Route mining procedure: Inputs and Outputs

Inputs.

- startNode — Neo4j node for the target Molecule
- compoundLabel — node label for molecules (e.g., 'Molecule')
- reactionLabel — node label for reactions (e.g., 'ChemicalEquation')
- relTypeInbound — inbound relationship spec (e.g., <REACTANT)
- relTypeOutbound — outbound relationship spec (e.g., <PRODUCT)
- configMap — optional bounds, e.g., {maxNumberReactions: k, nodeStopProperty: 'startingMaterial'}

Output. Stream of records, each record contains a single route as a sequence of relationships. NOCTIS converts each route to GraphRecord objects and aggregates them in a DataContainer.

Algorithm 1 `noctis.route.miner` (pseudocode)

```
procedure RouteMiner(startNode, compoundLabel, reactionLabel, relTypeInbound, relTypeOutbound, configMap)

Input validation:
   check startNode exists and has label compoundLabel
   check reactionLabel, relTypeInbound, relTypeOutbound are supported
   validate keys/values in configMap

Parse relationship specs:
   parse relTypeInbound → (type_in, dir_in)
   parse relTypeOutbound → (type_out, dir_out)

Route configuration:
   build RouteConfig from labels, relationship specs, and configMap
   bounds: maxNumberReactions (optional); stop: nodeStopProperty (optional)

Traversal evaluator:
   init SynTreeEvaluator with RouteConfig
   evaluator decides: stop conditions, AND/OR grouping, and bounds pruning

Initialization:
   create empty RouteLink rootRouteLink

Breadth-first traversal over bipartite layer:
   expand only via PRODUCT and REACTANT between Molecule and ChemicalEquation
   use custom relationship-uniqueness to avoid reusing the same relationship in a path
   use a relationship expander driven by parsed (type, direction) pairs
   at each step, SynTreeEvaluator:
      collects relationships into current AND segment (RouteLink)
      starts a new RouteLink when an OR Molecule is encountered
      prunes paths that violate depth or stop constraints

Collect partial segments:
   gather all RouteLinks produced by the evaluator
   if none and rootRouteLink non-empty, include rootRouteLink to the collection of RouteLinks

Group by root:
   group RouteLinks by their origin (RouteLinks starting node) using RouteLinksGrouper

Assemble complete routes:
   initialize a queue of OR nodes (branching points)
   map each OR node → list of what is going to be routes that end at that OR node. In the beginning, orRoutes map has empty values and the keys are OR nodes.
   for each RouteLink having as starting node startNode:
      if no OR nodes present in RouteLink: add the RouteLink as a final route
      else, add it under each leaf OR node in orRoutes map
   While the queue is not empty:
         pop an OR node from the queue
         for each RouteLink starting with that OR node:
            remove non-OR leaves (true leaves of the initial tree)
            extend the route from orRoutes map with each compatible RouteLink
            if in any of new routes no OR leaves remain: emit final route
            else: update orRoutes map and push new OR leaves back into the queue

Return: emit final routes as sequences of relationships (relationships)
   (NOCTIS wraps each in a GraphRecord and returns a DataContainer)
end procedure
```

Listing 1 Example Neo4j call

```
MATCH (paracetamol: Molecule {smiles: 'CC(=O)Nc1ccc(O)cc1'})
CALL noctis.route.miner(
  paracetamol,
  'Molecule',
  'ChemicalEquation',
  '<REACTANT',
  '<PRODUCT',
  {maxNumberReactions: 4, nodeStopProperty: 'startingMaterial'}
)
YIELD relationships
WITH relationships,
     [rel IN relationships | startNode(rel)]
   + [rel IN relationships | endNode(rel)] AS allNodes
RETURN apoc.coll.toSet(allNodes) AS nodes, relationships
```

Key components.

RouteConfig. Carries labels for molecule/reaction nodes, inbound/outbound relationship types and directions, and user bounds such as `maxNumberReactions` and an optional `nodeStopProperty` that halts traversal when present on a node.

SynTreeEvaluator. The traversal "brain": enforces bounds (maximum linear reaction length; early stop on `nodeStopProperty`); maintains AND/OR semantics (reaction nodes and single-provenance molecules are AND; multi-provenance molecules are OR); segments traversal into `RouteLinks` and starts a new segment at each OR branching point.

RouteLink. A minimal contiguous AND segment that must co-occur in a route (e.g., one reaction and all its required reactants, or a chain of such segments without passing through an OR molecule). Always starts and ends with `Molecule` nodes.

Custom relationship uniqueness. Tracks relationships that have already been seen on the current path to avoid revisiting the same relationship in cycles, preventing infinite loops while still allowing distinct paths through the same nodes if relationships differ.

Relationship expander. Restricts exploration to the bipartite core using only `PRODUCT` and `REACTANT` with the specified directions.

rootRouteLink. If the root target already forms a valid AND segment under tight bounds, it is retained so a single-segment route can be returned even when no branching is discovered.

RouteLinksGrouper & RouteAssembler. The former groups partial segments by their starting nodes; the latter performs OR-combination logic to produce complete routes, extending partial routes across queued OR molecules until all OR leaves are resolved.

orRoutes `orRoutes` is a working map used during route assembly. The map's keys are OR nodes, and the values are lists of partial route segments that currently terminate at that OR node, composed of `RouteLinks`. The assembler iteratively pops an OR node from a queue, retrieves corresponding partial routes from `orRoutes`, and combines each partial route with every compatible alternative `RouteLink` starting at that OR node. Newly formed routes that still end in OR nodes are recorded back into `orRoutes`,

while routes with no remaining OR leaves are emitted as finalized routes. When the route assembly is complete, this map should become empty again.

7.10 Test Tree for Route Mining

Figure 5 presents the test graph used to validate the route mining algorithm where all the chemistry is abstracted away (there is no real reactions and molecules). The Cypher script used to generate the graph, along with the expected output routes, is provided in the SI materials accompanying this article. The graph has been carefully designed to capture a variety of corner cases that may arise in real-world datasets, ensuring that the algorithm is robust and capable of handling such scenarios.

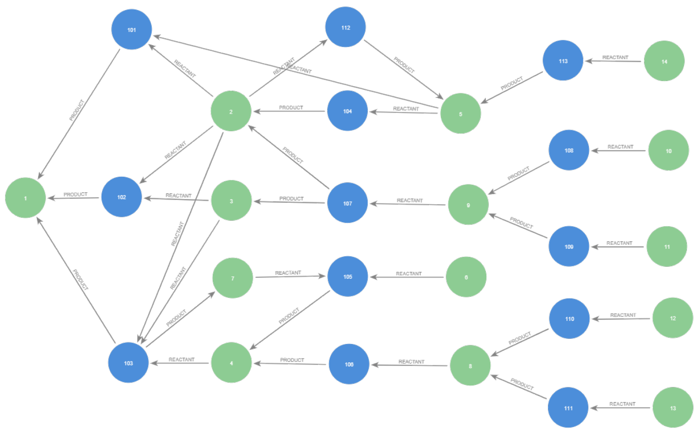

Fig. 5 Visualization of the test graph used for evaluating the route extraction algorithm

7.11 Workflow Class Interaction Diagrams

These diagrams illustrate the classes contained within each module and how they are invoked in the two main workflows: from file input to graph database construction, and from the graph database back to Python objects.

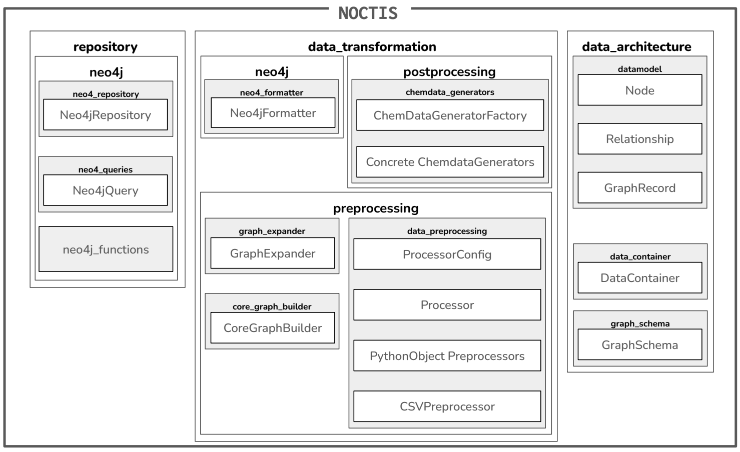

Fig. 6 Diagram of how main classes are organised in modules

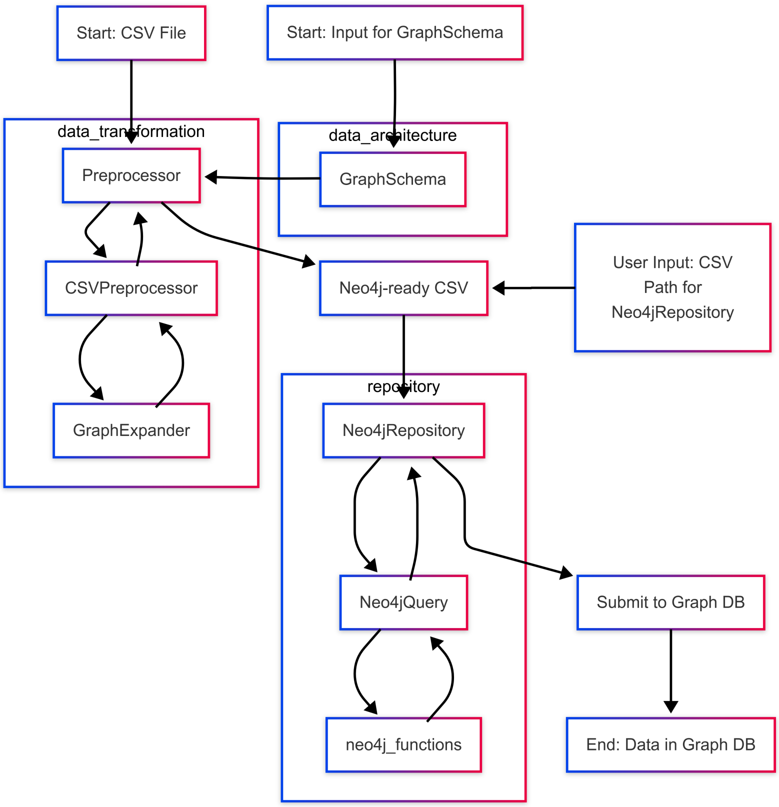

Fig. 7 Diagram of how classes interact when CSV is being put into a Graph Data Base

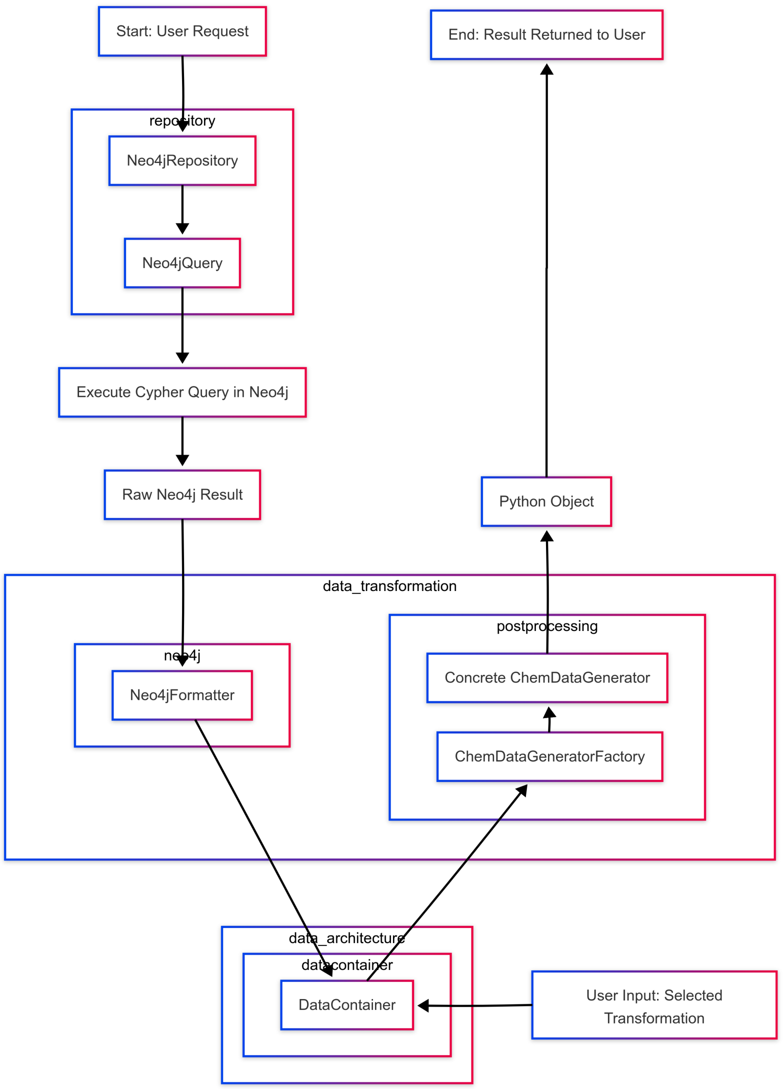

Fig. 8 Diagram of how classes interact when User queries a Graph DB and returns a Python Object.
